# Hyperhomocysteinemia: Metabolic Role and Animal Studies with a Focus on Cognitive Performance and Decline—A Review

**DOI:** 10.3390/biom11101546

**Published:** 2021-10-19

**Authors:** Hendrik Nieraad, Nina Pannwitz, Natasja de Bruin, Gerd Geisslinger, Uwe Till

**Affiliations:** 1 Fraunhofer Institute for Translational Medicine and Pharmacology ITMP, Theodor-Stern-Kai 7, 60596 Frankfurt am Main, Germany; n.pannwitz@t-online.de (N.P.); natasja.debruin@itmp.fraunhofer.de (N.d.B.); geisslinger@em.uni-frankfurt.de (G.G.); 2 Pharmazentrum Frankfurt/ZAFES, Institute of Clinical Pharmacology, Goethe University, Theodor-Stern-Kai 7, 60590 Frankfurt am Main, Germany; 3 Former Institute of Pathobiochemistry, Friedrich-Schiller-University Jena, Nonnenplan 2, 07743 Jena, Germany; uwe.till.erfurt@web.de

**Keywords:** hyperhomocysteinemia, vitamin B deficiency, dementia, disease models, animal

## Abstract

Disturbances in the one-carbon metabolism are often indicated by altered levels of the endogenous amino acid homocysteine (HCys), which is additionally discussed to causally contribute to diverse pathologies. In the first part of the present review, we profoundly and critically discuss the metabolic role and pathomechanisms of HCys, as well as its potential impact on different human disorders. The use of adequate animal models can aid in unravelling the complex pathological processes underlying the role of hyperhomocysteinemia (HHCys). Therefore, in the second part, we systematically searched PubMed/Medline for animal studies regarding HHCys and focused on the potential impact on cognitive performance and decline. The majority of reviewed studies reported a significant effect of HHCys on the investigated behavioral outcomes. Despite of persistent controversial discussions about equivocal findings, especially in clinical studies, the present evaluation of preclinical evidence indicates a causal link between HHCys and cognition-related- especially dementia-like disorders, and points out the further urge for large-scale, well-designed clinical studies in order to elucidate the normalization of HCys levels as a potential preventative or therapeutic approach in human pathologies.

## 1. Introduction

The metabolism of molecular groups with only one carbon atom (C1 metabolism) is part of the basic equipment of cells. Its products function as building blocks or as links in regulatory chains and are essential for the synthesis or completion of an enormous number of larger molecules. The large number and variety of products show that disorders of C1 metabolism can also lead to numerous symptoms and diseases. The present review aims to list disease groups or particular, mostly common diseases, for which the involvement of C1 metabolism and underlying pathological mechanisms are substantiated by relevant evidence, especially clinical studies including interventions. For this purpose, both the human and animal parts of the current review focus on the contribution of C1 metabolic disorders and hyperhomocysteinemia (HHCys) on cognitive performance and decline. In order to facilitate understanding, we prefixed a critical, general part with the subsequent five sections.

## 2. C1 Metabolism and HHCys

### 2.1. Reactions of the C1 Metabolism and Its Main Products

**Figure 1:**
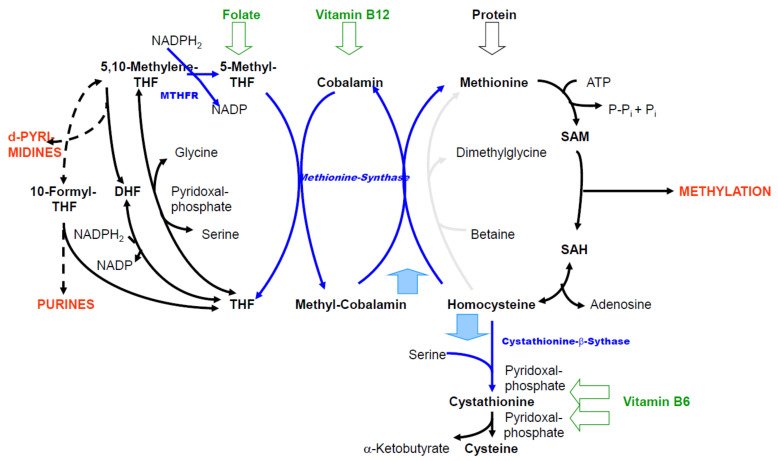
All essential reactions of the C1 metabolism: On the left side, the folic acid cycle is depicted, showing formyl and methenyl groups bound to tetrahydrofolate (THF), which is involved in purine synthesis (DNA, RNA), and methylene, involved in deoxythymidylate synthesis (DNA) and methyl groups that are required for the re-methylation of homocysteine (HCys) to methionine. On the right side, the methylation cycle is illustrated, including S-adenosylmethionine (SAM) methylations: nucleic acids, proteins, phospholipids, neurotransmitters, hormones, creatine and others. Histone protein, DNA and RNA methylations cause epigenetic regulation [1,2]. The vast majority of methylations originate from SAM [3]. More than 200 SAM-dependent methyltransferases are encoded in the human genome [4]. Red: products, intermediate reactions are omitted (dashed arrows); blue: reactions with enzymes for which genetic defects frequently occur or which catalyze reactions that can be reduced; green: necessary B-vitamins that cannot replace each other. The reaction catalyzed by methionine synthase needs two vitamins as cofactors at the same time; two light blue arrows: re-methylation of HCys (upwards), transsulfuration of HCys (downwards); MTHFR: 5,10-methylene tetrahydrofolate reductase, THF: tetrahydrofolate, DHF: dihydrofolate, SAM: S-adenosyl methionine, SAH: S-adenosyl homocysteine.

Figure 1 shows the reactions through which all C1 compounds are generated and it serves as a building block for the subsequent figures.

#### 2.1.1. Thermodynamic Features

(a) The reaction catalyzed by 5,10-methylenetetrahydrofolate reductase (MTHFR) proceeds almost completely unidirectional to 5-methyl-THF under normal metabolic conditions [5]. There is a reason for the so-called folic acid trap [6,7]: if there is a pronounced vitamin B12 deficiency, there is no re-methylation of HCys via methionine synthase (Figure 1). Even with sufficient folic acid intake, it accumulates as 5-methyl-THF potentially resulting in a deficiency in C1 compounds of the folic acid cycle.
(b) The reaction of S-adenosyl homocysteine (SAH) to HCys (by the SAH hydrolase) tends towards SAH formation [8]. In this way, the cellular HCys concentration is kept low under normal metabolic conditions.

#### 2.1.2. Special Features of the Enzyme Equipment and Kinetics

(a) In Figure 1, the re-methylation of HCys to methionine by betaine (betaine homocysteine methyl transferase) is marked in light gray, because the enzyme is only expressed in few tissues, such as liver and kidneys [6,9].
(b) The cellular concentrations of total HCys for most organs are 2–7 nmol/g wet weight [10]. Calculating with approximately 70% cell water results in concentrations of 3–10 µM. The K_M_ values for HCys of the initiating enzymes of re-methylation (methionine synthase) and transsulfuration (cystathionine-β-synthase) are 0.06 mM and approximately 10 mM [11] and thus, differ by three orders of magnitude. Serine, the second substrate of cystathionine-β-synthase (CBS), also has a high K_M_ value of 2 mM [12]. From this, it can be concluded that if there is an adequate supply with folic acid, vitamin B12 and B6, HCys is predominantly re-methylated.
(c) Transsulfuration is not possible in some tissues, because there is no expression of CBS (heart, vessels, lungs, adrenal gland, spleen, testes) or cystathionase (brain, adipose tissue) [11].
(d) The availability of sufficient SAM as a substrate for the majority of methylation reactions is a crucial function of C1 metabolism. In humans, 6–8 g SAM are synthesized daily [13]. Its synthesis is largely ensured by the effector functions of SAM and, at the same time, HCys metabolism is influenced, since SAM inhibits the MTHFR [14] and activates the CBS [15]. In cells that express both enzymes, when SAM levels rise (e.g., due to an abundant supply of methionine) this is irreversibly removed via transsulfuration. Owing to the high K_M_ value of CBS (cf. above) enhanced flux rate through transsulfuration is accompanied by an increase in cellular HCys concentration. When there is a deficiency of SAM, re-methylation of HCys is stimulated.
(e) SAH is a potent inhibitor of most SAM-dependent methylation reactions [16]. However, the consequences are different for individual methylations, as will be explained later.
(f) A special kind of methylation cycle arises from the ability of the methionine synthase to catalyze also protein-bound HCys, as in the case of the D4 dopamine receptor (D4). Stimulation of D4-bound methionine leads via D4-bound SAM to methylation of membrane phospholipids [17].

### 2.2. Principal Causes of C1 Metabolic Disorders

**Figure 2:**
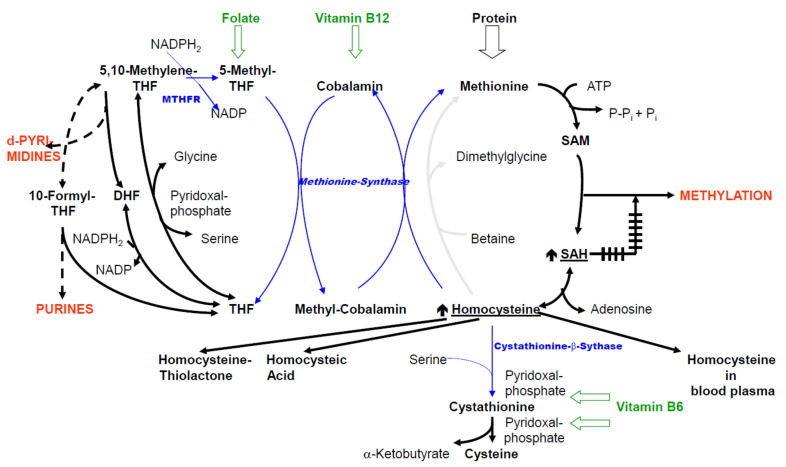
Adaption of the previous chart (colors and abbreviations: see Figure 1 caption): C1 metabolism in the case of vitamin deficiency or genetic enzyme variants; reductions in supply or turnover due to various causes are marked by thinner arrows; inhibitory effects are marked by the crossed-out arrow.

As illustrated in Figure 2, insufficient clearance with simultaneously constant production leads to increases in the levels of intermediates (HCys, SAH) and consequently, to the formation of new products, such as homocysteine thiolactone and homocysteic acid.

The following list of causes for disorders in C1 metabolism does not take into account genetic defects with prevalence ≤1:10,000, because they are too rare as disease triggers. Frequent causes can essentially be assigned to four groups (Table 1).

### 2.3. HCys as a Diagnostic Measurable Biomarker of Disorders

Numerous laboratory parameters for measuring individual causes of HHCys, such as molecular-genetic analyses, vitamin level measurements and others, only measure one parameter at a time and rarely provide information about the extent and severity of the disorder. They are not dealt here within this context.

Under consideration of Figure 2, it is clear that each of the causes discussed above must lead to an increase in cellular HCys. Concomitantly, this results in the increased formation of two secondary metabolites of HCys: homocysteine thiolactone and homocysteic acid. On the other hand, all human cell types investigated so far have transport systems that remove accumulated cellular HCys into the extracellular space, partly also against a concentration gradient [37,38,39]. Most of the elimination from plasma occurs in the kidneys. The clearance rate in healthy kidneys is remarkably constant, regardless of the plasma HCys level [39].

Plasma HCys is therefore a sensitive parameter for quantifying disorders in C1 metabolism. Compared to other parameters, it usually has higher sensitivity: plasma concentrations of HCys, folic acid, vitamins B12 and B6 were measured in more than 1000 participants in the Framingham Heart Study. For all three vitamins, the HCys level rose in the lower half of their reference range and was around 35% higher at the lower end [40]. Such studies were repeated several times with the (same) result of recommending HCys measurement, because the measurement of the three B-vitamins in plasma only allowed limited conclusions to be drawn about their cellular availability [27].

Plasma HCys levels show differences dependent on age and gender (measured in populations without folic acid supplementation) [27]: The values reach 10 µM at the age of 50 and 60 years in men and women and an increase to approximately 12 µM up to 80 years. The increase in older subjects is apparently also due to the lack of availability of the B-vitamins, since parenteral vitamin substitution lowers the level to that in middle age [41]. Depending on the responsible institution in European countries and in the USA, a level of 10–12 µM has been considered a threshold value. Moderate increase above these threshold values up to approximately 30 µM mark the range for human diseases listed in this part of the present review. These HCys levels are generally considered as a biomarker of disease and/or having a pathogenic effect. Some of the following incomplete findings from older studies, however, should be restrictively considered:

(a) Because HCys is transported out of the cells, the concentrations in the extracellular space and plasma do not have to correspond to those in the cells, which are responsible for the increased production. The liver is the main organ for HCys formation [39]. However, when HCys formation and export are stimulated, the cellular concentration in the liver remains relatively constant [10]. Cultured endothelial cells continuously export HCys into the medium and keep the cellular concentration at a significantly lower level [38]. In contrast, the addition of HCys to the medium (100 µM) leads to absorption and increases the intracellular concentration [38]. It can, therefore, be assumed that endothelial cells have only a small capacity to re-utilize HCys. Increase in HCys levels in plasma and extracellular space is not only effective at, but also in endothelial cells.
(b) Only free HCys is reactive. The ratio of free to protein-bound HCys is different intracellularly than in blood plasma [10]. For example, approximately 4.5 and 3 nmol/g wet weight for free and bound HCys were measured in rat liver, which exchange with a half-life in the range of seconds. The quotient of free/bound HCys is 1.47 for rat liver. For cerebrum and cerebellum, it is 2.72 and 17.81, respectively. Free HCys is exported [10].

Consequently, liver cells might keep constant HCys levels by exporting it to the plasma as a consequence of vitamin deficiency, whereas other cells take it up and highly increase their cellular concentration of free, reactive HCys.

For laboratory diagnostics, total HCys is measured in blood plasma. The major part is bound to albumin (approximately 80%), followed by disulfides with itself or cysteine (cf. Figure 4D). Free, reduced HCys makes up less than 2% [27]. If the level changes in the range of a normal or moderate increase in total HCys concentrations, this equilibrium between the fractions is restored very quickly [42]. With increased cellular HCys formation, free, reduced HCys is exported. Adjustment of the equilibrium in plasma inevitably forms reactive oxygen species (ROS). As shown in Figure 4E, every release of HCys is associated with the formation of radicals.

The use of plasma HCys as a marker of the detection and extent of a C1 metabolic disorder is only possible in patients without significant impairment of their kidney function. The kidneys play a major role in the elimination of HCys from the blood—not through excretion in the urine, which only accounts for about 1% of the daily amount of HCys produced [43]. This is only higher with extremely high HCys levels in plasma, for example with homocystinuria [44]. The renal clearance of HCys occurs mainly through re-methylation and transsulfuration [45,46]. Both pathways are reduced in renal insufficiency [46]. The kidneys are also the major organs for the clearance of SAH from plasma, both by filtration and by metabolism [47]. Kidney damage is typically accompanied by an increase in plasma HCys level. It not only affects the end stages of renal insufficiency, but also reflects the entire course of the disease: a meta-analysis of 41 studies with 2700 test subjects revealed a highly significant correlation between plasma HCys and the reciprocal value of the glomerular filtration rate [48]. It affects the entire range of the glomerular filtration rate [49]. Regardless of its cause, plasma HCys concentrations of 20–80 µM are measured in terminal kidney failure [50]. The increase in plasma HCys is caused by the kidney disease itself. The influence of confounders such as body mass index, plasma lipids, hypertension, smoking, diabetes mellitus and others could be excluded [51]. HHCys caused by renal insufficiency is the only form that mainly arises from reduced clearance of HCys and therefore differs primarily from all other forms of HHCys that result from increased HCys production as a result of disorders in cellular C1 metabolism.

### 2.4. Principal Pathological Mechanisms with Morbid Effects in C1 Metabolic Disorders

**Figure 3:**
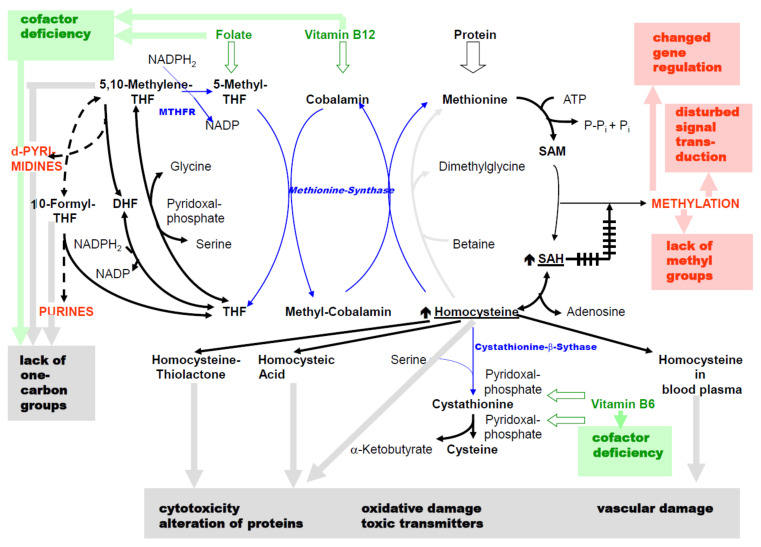
Adaption of the previous chart (colors and abbreviations: see Figure 1 caption): pathogenic effects of metabolites or products in the context of disorders in C1 metabolism.

Based on the previous charts, Figure 3 additionally highlights different pathogenic consequences of the disorders, which are subsequently discussed further.

Deficiency in one or more of vitamin B6, B12 and folate affects cofactors derived from them:

(a) The supply of C1 compounds from THF metabolites is reduced due to folic acid deficiency. This results in lack of nucleotides in energy metabolism and impairment of DNA and RNA synthesis. There is also impairment of mitotic rate.
(b) In addition to reduced HCys transsulfuration, vitamin B6 deficiency causes inhibition of numerous pyridoxal phosphate-dependent reactions in amino acid metabolism.
(c) Vitamin B12 deficiency leads to the accumulation of HCys.

The genetic variants for MTHFR and CBS, as well as acquired causes of inhibition of these enzymes, also lead to accumulation of HCys. The increase in cellular HCys levels also leads to the accumulation of SAH, which is a strong inhibitor of methylation reactions [16]. Consequences are: lack of methyl groups for syntheses, altered methylation of DNA and histones leading to disturbed epigenetic gene regulation, impairment of signal transduction when particular elements, e.g., kinases and phosphatases, are regulated via methylation [52].

As described above (cf. Section 2.1.2), the SAM/SAH as substrate/inhibitor quotient could regulate the overall activity of numerous methyltransferases, meaning increased or decreased methylation of all substrates. However, the regulating effect of the SAM/SAH quotient is differentiated by various mechanisms:

(a) The K_M_ values for SAM and the K_I_ values for SAH are different for individual methyltransferases and differ between the various enzymes by almost three orders of magnitude [52,53]. A changed SAM/SAH quotient can either do nothing at all, e.g., if the enzyme continues to work in the V_Max_ range, or result in changes in methylation.
(b) There are “buffer reactions” without metabolic effects, such as the methylation of glycine to sarcosine by glycine-N-methyltransferase, which regulates the SAM concentration [3].
(c) The cellular concentrations of SAM and SAH respond to changes in the intake of vitamin B6, B12 and folate, but evidently vary in different tissues and vary in the individual developmental stages of the organs [52,54].

**Figure 4:**
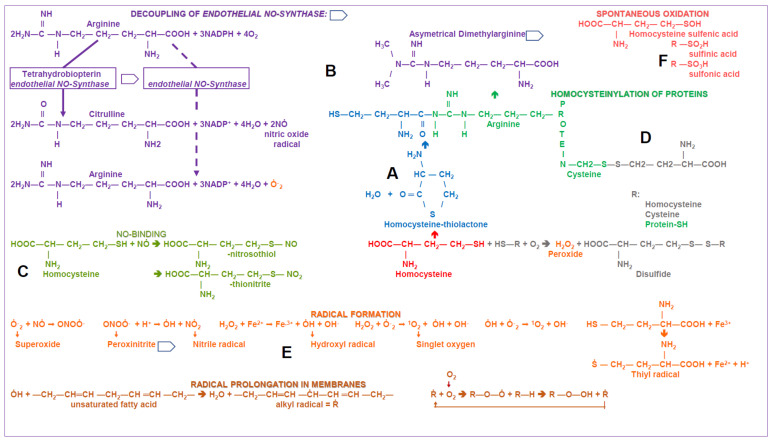
Homocysteine pathology; presentation of the most important possible reactions of HCys resulting in pathological effects; color differentiation and capital letters allow assignment to the different mechanisms and their effects: (**A**) In contrast to cysteine, HCys has a highly reactive sulfhydryl group, e.g., it can form a ring shape in the case of homocysteine thiolactone, which accounts for approximately 1/10 of the free HCys in blood plasma and can bind to lysine or arginine residues of proteins via a peptide bond (N-homocysteinylation) [55]. (**B**) In the latter case, asymmetrical dimethylarginine is released during the destruction of the protein, which decouples the endothelial nitric oxide (NO) synthase, so that it produces the superoxide anion instead of NO [56]. NO and the superoxide anion form peroxynitrite, which further contributes to decoupling of the enzyme complex [56]. (**C**) HCys itself can bind NO and thus inactivate it [57]. (**D**) HCys forms mixed disulfides with cysteine residues of proteins, called S-homocysteinylation, which can lead to functional impairments [58]. (**E**) HCys generates hydrogen peroxide via disulfide formation, from which all important ROS and radicals can arise [59]. (**F**) The spontaneous oxidation of the sulfhydryl group results in homocysteic acid (homocysteine sulfonic acid) with an agonistic effect on N-methyl-D-aspartate (NMDA) receptors [60].

In addition to being a biomarker of disorders, the increase in HCys levels can also have direct cytotoxic effects (Figure 4).

The increase in plasma HCys leads to vascular damage through covalent binding to proteins, formation of ROS and, as described before, inactivation of the vasodilator NO [57,61].

#### Experimental Use of Methionine or HCys

When working with cell cultures, in animal experiments and in humans, HCys is often applied directly, as further described in the second part of the review (cf. Section 4.1). The same applies to methionine, which is used in exercise tests in humans to temporarily increase plasma HCys concentration [62,63,64]. Physiologically, both amino acids occur only as L-enantiomers. The additives from commercial batches are, unless stated otherwise, racemates from L- plus D-form. Their effects are usually equated with those of the physiological enantiomers. This is not justified, because enzymatic reactions or receptors can be stereospecific and therefore, spatial orientation might play an important role:

(a) In humans, D- and DL-methionine show only 30% and 65% effectiveness, respectively, compared with L-methionine regarding the nitrogen balance [65].
(b) In chicks, D-HCys is only re-methylated to methionine to about 25% via methionine synthase, compared with L-HCys [66].
(c) In a methionine-deficient diet, L-HCys can replace 65% of the growth-promoting effect of L-methionine via this reaction, but D-HCys only 7% [67].
(d) Of the spontaneous oxidation products of HCys (Figure 4F), only L-homocysteine sulfonate and D-homocysteine sulfinate are selective activators of NMDA receptors, but not the D or L-enantiomers of the two acids [60].

### 2.5. Homocystinuria as a Result of an Existing Homozygous Defect in CBS—Witness of HCys Pathology

The pathogenic effects of disorders in C1 metabolism are usually complex. As stated earlier, HCys, resp. HHCys, has a biomarker function of these disorders. It remains to be seen, however, whether HCys is only a biomarker of disease or whether it is causally involved. For common disease groups, such as atherosclerosis and cerebral diseases, a causal involvement in the pathogenesis is plausible. Clinical intervention studies, however, frequently showed heterogeneous findings and resulted in assigning HCys only the role of a biomarker. The ambiguity of the study results is often due to study design, which is unsuitable for clarification of the question about causality. We, therefore, analyzed the studies from this point of view (cf. Section 3). In the following, however, an attempt will first be made to look at the pathological effects and symptoms of a disease with obviously isolated HHCys—without significant other disorders in C1 metabolism. These should then be referred back to HCys as a pathogenic agent. Corresponding conclusions can be drawn from analogies to common diseases. At CBS defect, primarily only the transsulfuration of HCys fails (Figure 2), with the consequence of excessive increase in plasma HCys of more than 100 µM and concomitant homocystinuria [44]. HCys is apparently the decisive pathogenic agent in this disease:

(a) There are no other causes of disorders in C1 metabolism, such as a vitamin deficiency.
(b) A suspected defect-related deficiency in cysteine or glutathione cannot be proven. Concentrations in plasma and urine correspond to those of controls [68,69].
(c) CBS also catalyzes the formation of hydrogen sulfide (H_2_S), which may be diminished. There are, however, two further enzymes that catalyze H_2_S formation from cysteine: cystathionine-γ-lyase and 3-mercaptopyruvate sulfurtransferase [70]. Moreover, HCys was found to upregulate cystathionine-γ-lyase in cardiomyocytes and also in vivo (*Cbs*+/− mice), the enzyme was upregulated [71]. Furthermore, even in the absence of pyridoxal-5′-phosphate, brain homogenates of CBS-knockout mice produced H_2_S levels from cysteine similar to those of wild-type mice by 3-mercaptopyruvate sulfurtransferase in combination with cysteine aminotransferase [72].

If left untreated, the disease is therefore to be regarded as a “key witness” for frequent HCys-associated diseases, because most of the organs and clinical symptoms affected are also found in common diseases, that are listed subsequently and for which a prophylactic or therapeutic lowering of HCys level is recommended (Table 2).

Since HCys must primarily have an effect on the disease in this defect, the analogies to the common diseases result in clear indications of a function of HCys as a pathogenic agent and thus a high level of plausibility for a causal role of this risk factor in the development of the disease. It affects a relatively large number of common diseases, which is probably due to the (untreated) lifelong effects of high HCys levels, including the developmental years.

## 3. Diseases in which C1 Metabolic Disturbances and HHCys Are Significantly Involved in the Pathogenesis

One aim of this review is to give an overview of human disorders that are discussed to be causally affected by elevated HCys levels. As the current review focusses on the impact of C1 metabolic disturbances, especially HHCys, on cognitive performance and decline, findings of relevant human studies are subsequently outlined (Table 3). The table mainly provides a summary of other, already existing reviews on this particular topic. Nevertheless, we also reviewed evidence on other indication areas, which are summarized in Appendix A.

## 4. Animal Studies on HHCys—Literature Search Results and Discussion

Based on the relevance of HHCys in humans, which has been teased out in the previous sections, this review indicates the need for adequate animal models for HHCys. Due to the diversity of HCys-related pathologies in humans and the large number of investigations in experimental animals, also this second part of the review focusses on cognition-related investigations of HHCys. Our goal was to collect, summarize and assess findings of various relevant animal studies in order to finally answer the question: “What is the current consensus of preclinical evidence on the impact of hyperhomocysteinemia on cognitive performance and decline?”.

In general, adequate animal models attempt to simulate a human disorder as comprehensive as possible. However, there is always a gap between the model and the human pathology, which is in most cases far more complex. Several strategies have been applied in order to induce HHCys in animals, providing pros and cons regarding the simulation of a disturbed HCys metabolism. The choice of an appropriate model should depend on the particular research question and the increase in HCys levels that the researcher aims to induce. With respect to animal research on HHCys, the following two sections focus on the analysis and discussion of the results from our systematic literature search. In Appendix B, all included animal studies are summarized (Table A13 and Table A14) and more information on the literature search strategy, the analysis of average HCys blood levels, as well as behavioral cognitive-related outcomes are provided.

### 4.1. HHCys Induction Methods in Animal Models

**Figure 5:**
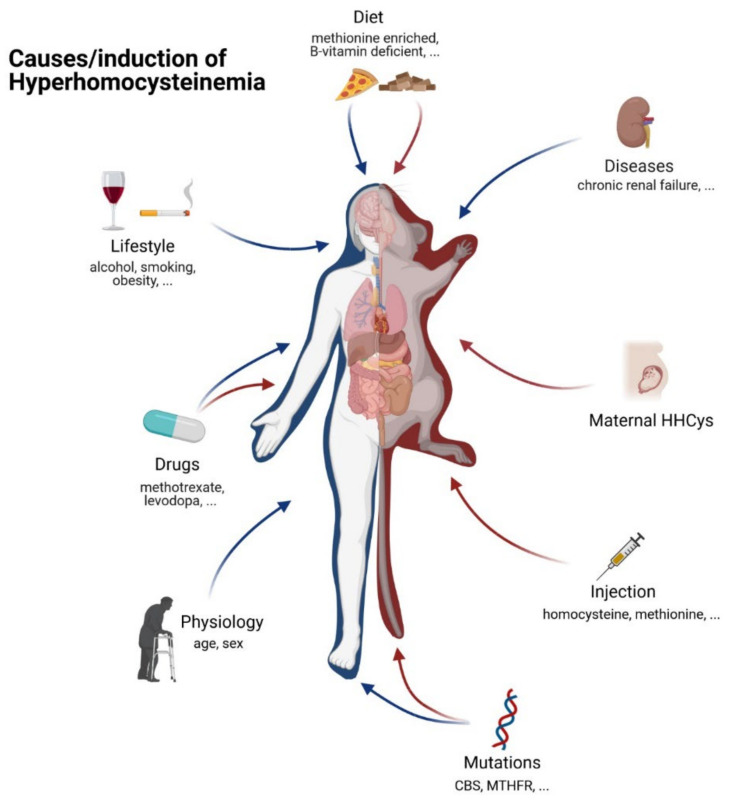
The most common causes of HHCys in humans (blue) and induction methods in animal models (red); created with BioRender.com.

Different factors have been determined as culprits in terms of the development of a hyperhomocysteinemic state in humans and therefore, served as targets for artificial manipulation in experimental animals (illustrated in Figure 5).

**Figure 6:**
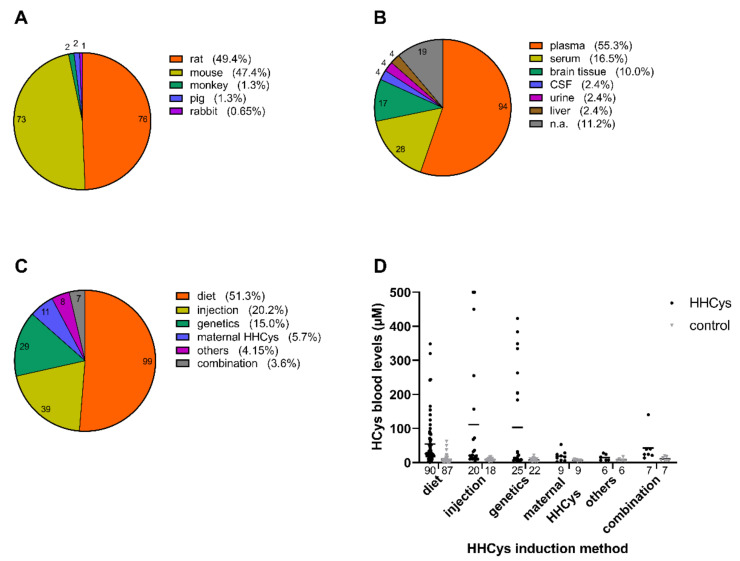
Average HCys levels and prevalence of different parameters, resulting from the analysis of the reviewed animal studies (numbers in the pie charts and at the bottom of the bars indicate the absolute amount of underlying studies): (**A**) animal species (154 cases in 154 studies in total); (**B**) biological matrices (170 cases in 154 studies); (**C**) HHCys induction methods (193 cases in 154 studies); (**D**) HCys elevation per induction method (every included study is considered as n = 1); since this is no meta-analysis according to the PRISMA guidelines, it should be considered semi-quantitatively; due to the large variation in individual studies, this panel should be used as a reference only; further methodological details are provided in the appendix of this review; created with GraphPad Prism 8 (San Diego, CA, USA).

As discussed in the first part of the review, HHCys is the result of either increased formation or decreased degradation of HCys, as well as decreased elimination due to impaired renal function. However, renal failure does not play a relevant role as an induction strategy of HHCys in animals, as it would display an unspecific method probably resulting in phenotypical artifacts. Relevant HHCys induction strategies, resulting from the literature analysis we conducted, are depicted in Figure 6C and subsequently described in-depth. As expected, rodent species played a pivotal role in animal experimentation towards HHCys (Figure 6A). In the reviewed animal studies, HCys levels in various biological matrices, such as blood, cerebrospinal fluid (CSF) and urine, as well as different tissues, e.g., brain and liver tissue, have been reported (Figure 6B). Average blood HCys levels (plasma, serum) are depicted in Figure 6D in order to show the HCys increase for each induction method.

#### 4.1.1. Dietary Induction

The most prominent strategy to induce HHCys in animals (cf. Figure 6C) is the dietary manipulation of different “players” in the C1 metabolism. On average, diets are fed for approximately three months. However, the duration of intake to build up HHCys is strongly dependent on the exact experimental diet. A feeding period of eight weeks is the most common duration in the reviewed studies. As indicated in Figure 1, a pivotal role belongs to several vitamins of the B series, affecting both the transsulfuration pathway and re-methylation of HCys. For that reason, diets deficient in B-vitamins, especially B6, B12 and folate, are a common option among the dietary HHCys induction methods (e.g., [78]). Additional supplementation with a sulfonamide antibiotic may further increase plasma HCys by inhibiting microbial folate synthesis in the gut [79,80]. In few trials, riboflavin (B2) and choline were also depleted from the chow (e.g., [81]). Vitamin B2 contributes to the catalytic functionality of the MTHFR and choline, a precursor of betaine and formerly known as vitamin B4, is another important methyl donor and therefore, also relevant for the homeostasis of HCys levels [82].

Additionally, plasma levels can also be elevated by excess consumption of L-methionine (e.g., [83], cf. Section 2.4). As the supplementation of chow or drinking water with methionine is another reliable method to induce HHCys in animals, this method was applied equally often as B-vitamin restriction. Interestingly, a combination diet of both B-vitamin deficiency and methionine supplementation did not additionally increase plasma HCys, but even lowered the levels compared to a diet with normal methionine content and a lack of B-vitamins [84]. According to the authors, this attenuation might be explained by an allosteric activation of the enzyme CBS by SAM [85]. However, similar investigations did not confirm this finding [86].

HCys levels can also be elevated by directly feeding the animals with HCys itself (e.g., [87]) or methyl group acceptors, interfering with the C1 metabolism. One example is guanidinoacetic acid, which is methylated to creatine and, for that reason, consumes a large portion of methyl groups provided by SAM [88]. In consequence, higher levels of SAH, and subsequently HCys, are built in the re-methylation cycle. Rarely, also nicotinic acid was used to elevate HCys levels [89]. Figure 6D shows that the entirety of the aforementioned dietary induction methods resulted in a mean blood HCys level of about 54 µM (versus 8 µM; control), which might be classified as a moderate HHCys.

#### 4.1.2. Parenteral Induction

The parenteral administration route is an alternative to diets in order to induce HHCys in experimental animals. In most cases, HCys is injected subcutaneously or intraperitoneally (e.g., [90]). In contrast to ad libitum dietary approaches, where the special chow is permanently offered to the animals, the frequency of injections is a major variable additionally to the dosage with respect to the chronicity of the resulting HHCys. The issue of separate injections was overcome by some researchers, who made use of osmotic minipumps in order to constantly infuse HCys [91,92]. However, since a major part of the reviewed trials reported acute HCys data, our analysis revealed a high mean level of about 111 µM (versus 8 µM; control), which indicates a severe HHCys (Figure 6D). Instead of directly administering HCys itself, few studies reported the administration of its metabolites homocysteine thiolactone [93] or homocysteic acid (HCA) [94], as well as the injection of drugs or L-methionine in order to increase HCys levels [95]. Despite of differing administration routes, the mechanisms underlying the induction of HHCys do not differ between peroral and parenteral protocols.

#### 4.1.3. Genetic Induction

Genetic animal models for HHCys are based on mutations in genes encoding for different enzymes that play central roles in the C1 metabolism (cf. Section 2). One of the most prominent enzymes in this context is CBS, which catalyzes the first step of the transsulfuration pathway in a vitamin B6-dependent manner. Reduced CBS functionality is responsible for decreased degradation of HCys to cystathionine and hence HCys elevation. Severe phenotypes were observed in experimental animals harbouring homozygous mutations in the *Cbs* gene, often leading to early death due to extremely high HCys levels (e.g., [96]). Due to limitations, especially lethality, in the investigation of homozygous (*Cbs−/−*) models, heterozygous (*Cbs+/−*) models were introduced (e.g., [97]) in order to enable comparison of biochemical and behavioral effects of less severe HHCys with wild type control (*Cbs+/+*).

Another prominent enzyme in the metabolism of HCys is the MTHFR, which enables an essential preliminary working step for the subsequent re-methylation of HCys by the vitamin B12-dependent methionine synthase. Because of the relevance of mutations in human pathology, genetic manipulation of the *Mthfr* gene has equally been utilized to introduce homozygous (*Mthfr−/−*) and heterozygous (*Mthfr+/−*) animal models for HHCys (e.g., [98]). In comparison to CBS-based models, MTHFR mutations only result in a mild to moderate elevation of HCys levels, which is also translationally relevant as human data, likewise, show higher HCys in the case of impaired CBS function than impaired MTHFR function [99].

In addition to CBS and MTHFR, other enzymes are summarized below, which are directly or indirectly involved in HCys homeostasis. Since these enzymes only play a minor role as HHCys induction method in animals, we summarized them as “others” in the literature analysis (Table A13 and Table A14). One example is cystathionine-γ-lyase (CTH), also known as cystathionase, which is involved in the transsulfuration pathway by catalyzing the conversion of cystathionine to cysteine in a vitamin B6-dependent manner. Although mutations in the *Cth* gene might even be more prevalent than in the *Cbs* gene in humans [100], CTH-based models are scarcely utilized in animal research so far, although *Cth−/−* proved to highly elevate serum and CSF HCys levels [101]. Another example is the betaine homocysteine methyl transferase (BHMT), of which homozygous (*Bhmt−/−*) and heterozygous (*Bhmt+/−*) forms were used in order to induce mild HHCys [102]. BHMT is involved in the reduction of HCys levels in a vitamin B12 and folate independent manner, by re-methylating HCys to methionine using betaine. Similar to BHMT, genetic modification of the methionine synthase reductase (encoding gene: *Mtrr*), which is responsible for the activation of the methionine synthase, resulted in slightly increased plasma HCys [103]. The average HCys level for genetic induction methods (Figure 6D) was approximately 103 µM (versus 7 µM; control), which might be classified as severe HHCys and therefore, equally to the human context, genetically-induced HHCys reflects higher levels than dietary-induced HHCys.

#### 4.1.4. Impact of Maternal HHCys

About 6% of the reviewed trials were summarized under the term “maternal HHCys impact” (e.g., [104]), comprising all the studies that focused on HCys levels in newborn pups. For several reasons, this is a special strategy for the induction of HHCys in animals. The primary HHCys induction is not applied in the pups, but in the dams, using one of the methods described in the paragraphs above and it can be applied even before pregnancy, during pregnancy or until weaning. Thus, it is possible to expose the offspring to elevated HCys levels via trans-placental transmission, resp. lactation, even during the very early stages of development. An average blood HCys of about 19 µM (versus 6 µM; control) was measured in pups (Figure 6D).

#### 4.1.5. Combinatory and Other Induction Methods

A small percentage of the reviewed studies utilized combinatory approaches of the aforementioned methods to induce HHCys, mainly by combining dietary and genetic models. In addition, researchers made use of more “exotic” strategies, such as the manipulation of parameters that are termed “lifestyle factors” in the human context. Xu and colleagues induced obesity by feeding a high fat diet und measured significantly elevated HCys in the hippocampi of the mice [105]. An elevation of plasma HCys of approximately 60% has been reached by the application of chronic unexpected mild stress to the animals [106]. Both of the aforementioned effects were apparently driven by a reduced CBS activity. An even higher increase in plasma HCys was observed in mice, fed with alcohol for several weeks [107]. This increase probably resulted from the interaction of ethanol with essential enzymes in re-methylation cycle of HCys [108].

Equally, physiological parameters, especially age, have been shown to contribute to elevated HCys in plasma and brain tissue [97,109]. Furthermore, a heterogeneous compilation of different animal treatment options has been proven to lead to HHCys. These range from inhalative N_2_O [110], peroral AlCl_3_ [111,112] and γ radiation exposure [113] to mechanical olfactory bulbectomy [114]. Of particular interest in the context of HHCys and dementia-like disorders is the potential impact of amyloid-β (Aβ) on HCys levels, as shown by infusing Aβ in rats [115]. Aβ pathology is a central hallmark of Alzheimer’s disease, which is the leading cause for dementia, accounting for approximately two thirds of all cases [116]. In a recent kinetic study in an amyloid-based mouse model for Alzheimer’s disease, we confirmed a significant effect of Aβ pathology on HHCys [117]. Finally, HHCys can also be pharmacologically induced in animals, e.g., by using the folate antagonist methotrexate ([118]). A comprehensive list of HCys level-modifying drugs has been provided in a previous review [27].

A limitation of the literature analysis in this review is that the assessment of HHCys has to be considered semi-quantitative (Figure 6D) and not as a quantitative meta-analysis, since not all aspects of the PRISMA guidelines for systematic reviews are fulfilled, as further explained in Appendix B.

### 4.2. HHCys Impact on Cognition in Animal Models

Based on the potential relevance of C1 metabolism disturbances for human health (cf. Section 3) and the variety of different animal models of HHCys, a huge amount of preclinical evidence has been accumulated by now. For that reason, the current review particularly focusses on the potential association of HHCys and impaired cognitive performance, resp. cognitive decline and dementia. In contrast to the early stages of HCys research, mainly focusing on cardio-vascular phenomena, HCys research in the context of neurodegeneration and cognitive abilities mainly gained increasing importance during the past 20 years.

**Figure 7:**
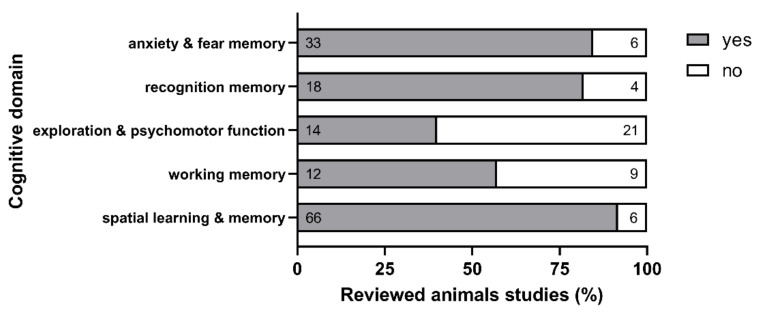
Impact of HHCys on cognitive performance; data resulting from the analysis of the reviewed animal studies (numbers in the bars indicate the absolute amount of underlying studies); different cognitive domains were analyzed: anxiety and fear memory (39 studies), recognition memory (22), exploration and psychomotor function (35), working memory (21), spatial learning and memory (72); in total, cognitive tests were performed in 102 of the reviewed animal studies; further methodological details are provided in the appendix of this review; created with GraphPad Prism 8 (San Diego, CA, USA).

Regarding the available literature until 2020, preclinical evidence suggests a causal link between HHCys and cognitive performance and cognitive decline. As summarized in Table A13, the vast majority of the reviewed animal studies (approx. 9 of 10 studies) revealed an impact of HHCys, meaning that, in these studies, at least one of the conducted behavioral tests showed significant effects following an elevation of HCys levels. In order to enable a consistent analysis, the plethora of behavioral tests in the reviewed studies was summarized in different cognitive domains (Figure 7). Further methodological information is provided in Appendix B. Interestingly, not all of the investigated behavioral domains were equally affected by HHCys: spatial memory, recognition memory and anxiety were affected in about 80–90% of the reviewed studies, whereas only approximately half of the relevant trials reported an impairment of working memory or psychomotor abilities through increased HCys. A potential explanation is an altered susceptibility of the different underlying brain areas to HCys-related damage. As shown previously, hippocampal structures are more vulnerable to HCys [119] and HCA [120] than cortical structures. Explorative behavior and psychomotor abilities, which are primarily associated with brain areas such as cerebellum and different cortical regions, indicated a lower susceptibility to HCys-related damage than spatial learning and memory, which is primarily associated with the hippocampus [121]. The hippocampal formation is implicated in both (spatial) working memory (short-term memory), and spatial learning and memory (long(er)-term memory). However, short-term working memory appears to be less affected by HCys-driven damage (Figure 7).

With regard to the frequently used HHCys induction method via B-vitamin deficient diet and its resulting effects on behavioral outcomes, the central question remains, whether the impairment of cognitive performance is actually a consequence of HHCys or an artifact due to the lack of essential B-vitamins. To elucidate this topic, we separately assessed all the reviewed studies, in which an elevation of HCys levels was reached only by parenteral administration of HCys itself (e.g., [122,123]). In these studies, no further manipulation of the C1 metabolism of the animals was undertaken. Nearly all of them reported a cognitive deterioration induced by the injection of HCys. Consequently, HHCys might be considered as a stand-alone risk factor for cognitive decline, independent of a restriction of B-vitamins.

HCys as a potential risk factor for Alzheimer’s disease (AD) in particular, was the subject in numerous of the reviewed studies. In the case of AD, most of the available preclinical models are based on genetic modifications relevant in amyloid metabolism and rather simulate the early onset form of the disease, which accounts for only 1% of AD patients [124]. The simulation of a more comprehensive AD-like phenotype inspired by the more prevalent late onset form of AD, might be reached by an additional induction of HHCys in these mouse models, as elevated HCys levels are common in the elderly [125].

The vast majority of research articles and review papers in the field focus on potentially detrimental effects of HCys itself. However, it should be emphasized that metabolites of HCys, such as HCA, might be the actual culprits. Previous findings indicated that higher concentrations of HCys are needed to suppress the activity of neuronal circuits to the same extent as HCA does [126]. Not HCys itself, but HCA seems to be responsible for toxic calcium influx into neurons [127]. Furthermore, investigations at ionotropic NMDA and metabotropic glutamate receptors revealed that ROS are produced to a higher extent by HCA than by HCys [128]. In a recent human study, Hasegawa and colleagues considered HCA as an early diagnostic marker of mild cognitive impairment and as more relevant than HCys in this context [129]. Earlier, it has been shown that treatment with an anti-HCA antibody attenuated cognitive impairment in the 3xTg-AD mouse model [130]. Interestingly, despite the aforementioned findings in humans and animals, HCA merely played a role in few of the reviewed trials, which are highlighted in Table A13 and Table A14.

Finally turning back to evidence derived from human trials, findings are not as clear as described for animal studies at the beginning of this section. This topic was not further addressed in our literature search, as there are other recent reviews available, concentrating on the equivocal role of HHCys and B-vitamins in the context of cognitive abilities in humans [75,131,132,133]. Both data pro [134,135,136,137,138,139,140,141,142,143,144] and contra [145,146,147,148,149,150,151] a causal link have been reported, systematically assessed and discussed through many years. We have the impression that, also in the clinical context, the sum of human studies predominantly strengthens the aforementioned causality, however, there is a larger portion of articles reporting negative results, compared to the field of animal studies. The design of some of these studies has been under criticism [152]. The gap in ambiguity between the clinical and preclinical field can probably, at least in parts, be explained by a publishing bias. Publishing bias is a common phenomenon, particularly in preclinical research, meaning that studies yielding positive results are more likely to be published than negative or null results [153,154,155]. Furthermore, overstatement of findings can occur due to lack of procedures such as randomization, blinding and appropriate power calculation [156,157,158]. Approaches such as pre-registration, which can help in reducing publishing bias, are more commonly applied in human studies than in animal research [155]. At present, there are preclinical initiatives attempting to raise awareness of complying with consistent quality parameters in order to struggle bias and attenuated reproducibility [159,160].

## 5. Summary and Conclusion

In the first part of this review, we outlined and critically discussed the metabolic role and effects of C1 metabolism disturbances, especially HHCys. These seem to contribute to versatile human disorders of diverse indication areas, ranging from the metabolic and vascular area to psychological and cognitive disorders, as well as the reproduction system, bone fracture rate and others. Based on this, the need for adequate animal models for HHCys becomes clear, as they are crucial to better understand basic processes and pathomechanisms. With the help of a systematic literature search and a focus on the link between HHCys and cognition, we summarized studies of the aforementioned animal models and analyzed the findings with the aim to assess, whether the majority of animal studies indicates a tendency pro or contra a causative role of HHCys in cognitive decline. Regarding the entirety of the reviewed preclinical evidence, the vast majority of included studies (approx. 9 of 10 studies) reported an impact of HHCys on cognitive outcomes and therefore underpinned a potential role of HCys in this context. With respect to the clinical situation, this means that it is firmly recommended to conduct additional large-scale and well-designed human studies to elucidate, whether the normalization of HCys levels represents a valuable preventative or therapeutic approach in terms of HCys-related pathologies in humans.

## Figures and Tables

**Table 1:** Frequent causes of C1 metabolic disorders.

Cellular deficiency in one or more of the vitamins B6, B12 and folate:
(a) Insufficient intake via food: Vitamin B12 deficiency in vegetarians and vegans, who do not supplement vitamin B12 [18,19]. All three vitamins in elderly subjects, especially in nursing homes [18,20]. Pronounced folic acid deficiency in industrialized countries around the world [21,22]. It is the main reason for folic acid supplementation in more than 70 countries [23]. (b) Loss due to inadequate preparation, especially for folic acid [24]. (c) Increased need during pregnancy, lactation and hemodialysis. (d) Insufficient intestinal absorption: unspecific in celiac disease, inflammatory bowel diseases and resections, specific for B12 with intrinsic factor deficiency or auto-antibodies against parietal cells [25,26]. (e) Intracellular, metabolic causes, e.g., accumulation of 5-methyl-THF in the case of pronounced B12 deficiency (“folic acid trap”), leading to a deficiency of THF-dependent C1 compounds, despite adequate folic acid intake [6,7]. (f) Side effects of pharmaceuticals on absorption or metabolism of particular vitamins, e.g., anticonvulsant drugs, levodopa, metformin [27].
Common genetic variants in C1 metabolism:
(a) MTHFR: C677T point mutation in homozygous form (TT) in 12–15% of the European population, which can be compensated by adequate folic acid intake [28]. Combined occurrence of the heterozygous form (CT) with the heterozygous form (AC) of another point mutation—A1298C—is relatively common (approximately 25%) and can be associated with various disturbances [29,30]. (b) CBS: About 230 known mutations that are rarely homozygous. In the heterozygous form, they potentially occur in around 1% of the European population [31].
Lifestyle factors (the underlying mechanisms are often not clear or multifactorial and usually linked to their effect on plasma HCys level):
(a) Acquired reductions in the activity of enzymes, e.g., methionine synthase due to acetaldehyde in alcoholics [32]. (b) Cigarette smoking appears as an independent determinant of HCys levels, with an increase in approx. 1% per cigarette smoked [33]. (c) Relatively large amounts of coffee consumption are necessary to increase HCys [34].
Oxidative stress:
Particularly nitric oxide inhibits methionine synthase directly, as well as by binding cobalamin [35]. Consequently, increase in plasma HCys is accompanied by that of markers of NO formation, e.g., citrulline. In addition, methylmalonic acid strongly increases as NO inhibits cobalamin transport from cytosol into mitochondria [36].

**Table 2:** Clinical symptoms associated with a homozygous CBS defect in analogy to common HHCys-related diseases (green).

Vessels:
- Arteries: intimal thickening, media destruction, fibrous plaques, thrombosis
→ cf.: atherosclerosis and its complications
- Veins: deep leg vein thrombosis, embolism
→ cf. thrombosis, embolism
If left untreated, most patients die in childhood or adolescence from consequences of vascular damage: arterial and venous thrombosis, embolism, myocardial infarction and stroke
Central nervous system:
- Mental retardation, epilepsy
→ cf. cognitive impairment, dementia, depression
Skeleton:
- Marfanoid habit with arachnodactyly, bone deformities, osteoporosis
→ cf. increased fracture rate
Eyes:
- Lens dislocation and severe myopia, also possible cataract, optic atrophy and retinal degeneration
→ cf. retinopathies and macular degeneration

**Table 3:** Cognitive decline and dementia; left column: relevant HCys-associated pathomechanisms; right column: correlation analyses and information on clinical studies; citation of other reviews or meta-analyses is marked as Rev [citation], followed by the reported findings, without individual quotations.

**Hypo-methylation—Rev** [52]; also see Figure 3: HCys ↑ → SAM/SAH ↓ → hypo-methylation of the presenilin 1 gene → increased β-amyloid formation. Hypo-methylation of the enzyme protein phosphatase 2A → loss of activity for phosphate cleavage of protein tau → accumulation of over-phosphorylated protein tau in neurofibrils → deposition of neurofibrillary tangles.	**Clinical studies—Rev [73,74,75]:** Plasma HCys negatively correlates with the thickness of the medial, inferior temporal lobe in normal subjects; equally in Alzheimer’s patients (already lower baseline values). Meta-analysis (77 case-control studies, 33 prospective studies, 46,000 subjects):	
**Neurotoxicity—Rev** [73]; also see Figure 4E,F: HCys and oxidation products (homocysteic acid) activate NMDA receptors → excitotoxicity (cellular Ca^2+^ increase → activation of proteases and radical formation → cell death = neuronal degeneration). Increased formation of ROS → activation of NFκB → inflammatory reaction.	Plasma-HCys	Risk
	≥15 μM	3-fold for cognitive impairment
	≥14 μM	2-fold for Alzheimer’s dementia
	Of approx. 10 placebo-controlled intervention studies with the three B-vitamins (B6, B12, folate), only five meet the decisive criteria: primary preventive approach, increased HCys starting level, study duration of at least two years, adequate vitamin dosage, proven decline in cognitive parameters in the placebo group. Significant results of these studies in favor of the vitamins: reduction of the brain atrophy rate, mainly gray matter, significantly better values for dementia status, MMSE (mini mental state evaluation) and learning test. Positive influence of plasma omega-3 fatty acid level on the effect of the B-vitamins [76]. Patients in the Alzheimer’s prodromal stage benefit from multi-nutrients with B-vitamins and omega-3 fatty acids: significantly better dementia status. The effect correlates directly with the baseline MMSE value [77] → importance of early start of prevention!	

Brain tissue: no HCys transsulfuration and no re-methylation of HCys to methionine by betaine (cf. Section 2.1) high sensitivity to folic acid and vitamin B12 deficiency.

## Data Availability

Not applicable.

## Appendix A

Due to the large number of diseases, in which C1 metabolic disturbances and HHCys are potentially involved in the pathogenesis, these are subsequently presented in concise tables (Table A1, Table A2, Table A3, Table A4, Table A5, Table A6, Table A7, Table A8, Table A9, Table A10, Table A11 and Table A12) with two columns: on the left, we summarized relevant HCys-associated pathomechanisms and on the right, correlation analyses and information on clinical studies are provided. Diseases with a comparable pathogenesis are grouped and facts are briefly communicated with reference to previous sections of the review. Where available, recent reviews or meta-analyses are cited, marked as Rev [citation], followed by the reported findings, without individual quotations.

**Table A1:** Atherosclerosis and its consequences: cardiac ischemia, peripheral occlusion, cerebral ischemia.

Disease course over years or decades without symptoms. Elevated HCys values are pathogenetically involved. Clinical symptoms first become prevalent through complications of atheromatous plaques with a different pathogenesis, in which B-vitamins and HCys hardly play a role. **Initial endothelial cell damage—Rev** [61,161]; also see Figure 4A–C: Reduced formation and efficacy of NO → inadequate vasodilatation in response to atherosclerosis-promoting stimuli. After coronary angiographic localization of such functional restrictions, vascular constrictions can be found after years in patients with acute coronary syndrome [162]. HCys causes increased formation of ROS (see Figure 4E) → cell activation with increased formation of adhesion molecules and pro-inflammatory cytokines; cell damage; apoptosis. S-homocysteinylation of endothelial proteins → loss of function (see Figure 4D). N-homocysteinylation by HCys-thiolactone → cytotoxicity (see Figure 4A). With HCys, the cellular SAH concentration increases → altered DNA or RNA methylation (see Figure 3) → reduced expression of enzymes with an antioxidant effect. Decrease in the thromboresistance of the endothelial surface by promoting coagulation and inhibiting anticoagulant and fibrinolytic mechanisms. **Plasma lipids and white blood cells—Rev** [161]: Oxidation of LDL → uptake by white blood cells → promotion of foam cell formation. Increase in chemotactic motility of white blood cells. **Smooth muscle cells—Rev** [61]: Oxidative stress → activation of the transcription factor NFκB → proliferation. **Platelets—Rev** [61]: Increase in thromboxane A_2_ synthesis → promotion of reactivity and aggregation.	**Studies on the influence of plasma HCys concentration on atherosclerosis consequences:** Recording of the period until the onset of symptoms. Elimination of conventional risk factors. Meta-analysis from >70 case-control studies as well as prospective studies with >20,000 subjects: An increase in HCys of 5 µM results in a 33% increase in risk for ischemic heart disease and 59% for ischemic stroke [163]. Meta-analysis from 12 prospective studies with >9000 subjects: Lowering HCys by 3 μM results in an 11% decrease in risk for ischemic heart disease and 19% for ischemic stroke [164]. Peripheral occlusion: case-control studies, with significantly higher levels of HCys than controls [165]. Renal insufficiency: high HCys levels (cf. Section 2.3). Greatly increased risk for all consequences of atherosclerosis, which are the main causes of death [166]. **Prospective intervention studies—Rev** [167,168]: A total of 15 studies had the effect of at least one of vitamin B6, B12 or folate was compared with placebo. 11 of these studies were secondary preventive—*after* a clinical event such as myocardial infarction—the remaining 4 studies in renal insufficiency requiring dialysis → no primary prevention overall. Results: heterogeneous/controversial. For ischemic stroke only, 25% risk reduction with the three B-vitamins. Only one primary preventive intervention study: >20,000 subjects with hypertension received folic acid + ACE inhibitors (Enalapril) versus only ACE inhibitors for 5 years: 34% reduction in ischemic stroke, 20% reduction in the combination of stroke, myocardial infarction, cardiovascular death. Renal insufficiency: meta-analysis of a total of 3886 patients: Monotherapy with folic acid resulted in a significant reduction in cardiovascular endpoints by 15%; in patients without (additional) dietary folic acid fortification by 20% [169].

**Table A2:** Metabolic syndrome and type 2 diabetes mellitus.

With a comparable HCys level, oxidative stress is stronger than in controls [61], directly detectable in myocardial fibrils (bypass surgery): H_2_O_2_ production ↑, antioxidants ↓ [170]. Metformin therapy inhibits intestinal absorption of vitamin B12 and folic acid [171].	Plasma HCys level as in controls, but higher if nephropathy is present. HCys is more strongly associated with atherosclerosis and its consequences than in non-diabetes controls [172]. Primarily preventive, folic acid supplementation lowers HCys and insulin levels, reduces insulin resistance, normalizes glucose homeostasis and atherogenic plasma lipid profile [173]. Metformin therapy lowers B12 levels, increases HCys levels and intensifies diabetic neuropathy [174].

**Table A3:** Thrombophilia: venous thrombosis, embolism.

HCys causes a decrease in the thromboresistance of the endothelial surface by promoting coagulation and inhibiting anticoagulatory and fibrinolytic mechanisms [61].	**Meta-analyses of clinical studies**—[175,176]: An increase in HCys of 5 μM increases the risk of deep vein thrombosis by 60% (case-control studies) or 27% (prospective studies). Patients with deep vein thrombosis and pulmonary embolism have significantly reduced plasma folic acid and/or vitamin B12 levels. Intervention studies so far unsatisfactory. The combination of HHCys and factor V (Leiden) is multiplicative [177].

**Table A4:** Depression.

**Reduced transmitter formation**—**Rev** [52]; also see Figure 3: Folic acid and SAM necessary for the synthesis of serotonin, noradrenaline and dopamine, both directly as well as via the synthesis of tetrahydrobiopterin. Significant changes in patients with depression: increase in HCys (plasma), decrease in folic acid (plasma, erythrocytes, liquor), SAM (liquor) and metabolites of the 3 transmitters (liquor)	Case control studies: Plasma HCys >10 μM → doubling the risk of depression [178]. Intake of vitamin B6, B12, folate correlates negatively with the occurrence of depression (12 years observation period) [179]. Intervention studies: The three B-vitamins versus placebo in patients at risk of depression for 7 years: significantly lower frequency [180]. Therapy with antidepressants in combination with folic acid, 5-methyl-tetrahydrofolate or SAM: better effect than antidepressants alone [52].

Brain tissue: no HCys transsulfuration and no re-methylation of HCys to methionine by betaine (cf. Section 2.1) high sensitivity to folic acid and vitamin B12 deficiency.

**Table A5:** Autism—**Rev** [75].

HCys and oxidation products (HCA) activate NMDA receptors → excitotoxicity (cellular Ca^2+^ increase → activation of proteases and radical formation → cell death = neuron degeneration)**—**see Figure 4F	Case-control studies: significant deviations in plasma levels in autistic children: HCys ↑; vitamin B6, B12, folate ↓. Interventional study: folic acid supplementation lowers plasma HCys levels and reduces deficits in cognition, communication and social behavior.

Brain tissue: no HCys transsulfuration and no re-methylation of HCys to methionine by betaine (cf. Section 2.1) high sensitivity to folic acid and vitamin B12 deficiency.

**Table A6:** Pharmacotherapy of neurodegenerative diseases: Parkinson’s disease, epilepsy.

**Levodopa** is degraded by methylation → significantly higher HCys levels than in untreated Parkinson’s patients → increased risk of stroke, coronary artery disease, dementia and peripheral neuropathy [181]. **Anticonvulsants**, especially valproate, influence the metabolism of folic acid (inhibition of cellular receptors), vitamin B6 (increased degradation) and reduce betaine uptake → lowering of plasma level of the two vitamins and increase in HCys [182]—see Figure 2 Particular risk: carrier of the TT variant of the C677T mutation of the MTHFR—see Table 1	Substitution with vitamin B6, B12, folate lowers HCys levels [183]. Significantly more femoral neck and vertebral fractures [184] and brain atrophy **Rev** [185]. Pregnancy: 10-fold increased risk of abortion, malformations in 6–11% of newborns, often cognitive deficits [186]. Improvement after supplementation with folic acid and B6 [184] or folic acid and B12 [186].

Brain tissue: no HCys transsulfuration and no re-methylation of HCys to methionine by betaine (cf. Section 2.1) high sensitivity to folic acid and vitamin B12 deficiency.

**Table A7:** Peripheral neuropathy.

Most frequent cause: damage to the peripheral myelin protein-22 [187]. HHCys → methylation disorders (see Figure 3): Hypo-methylation of Arg_107_ of the basic myelin protein → loss of binding for acidic lipids → disrupted lamellar formation.	HCys ↑ causally affects neuropathies in: Parkinson’s disease under levodopa therapy, type 2 diabetes mellitus (especially with metformin therapy), chronic alcoholism. Supplementation with vitamin B6, B12, folate improves the symptoms [170].

**Table A8:** Pregnancy and childbirth.

Pregnancy changes laboratory parameters for assessing C1 metabolism: U-shaped course with a lowering of vitamin B6, B12, folate and HCys in plasma in early pregnancy. Only holotranscobalamin remains constant [188]; only HCys levels in the interval are meaningful. Threshold for women of childbearing age: 9 μM	Evaluation of >14,000 pregnancies: HCys level in the interval >8.9 μM → significantly increased risk of preeclampsia, premature birth, stillbirth, low birth weight, neural tube defects [189].
**Pregnancy complications: preeclampsia—Rev** [190]**, abortion** **Preeclampsia** and cardiovascular diseases are essentially **one** entity—women with 2–3 preeclampsia episodes: significantly elevated laboratory parameters (HCys, von Willebrand factor, fibrinogen, insulin, total cholesterol, VLDL, triglycerides). Significantly increased risk of hypertension, coronary artery disease, stroke, venous thromboembolism, type 2 diabetes mellitus, cardiovascular mortality	
**Preeclampsia:** decoupling of the endothelial NO synthase (see Figure 4B) → significant reduction in endothelium-dependent vasodilatation. **Abortion:** HCys level >18 μM: significantly reduced vascularization of the villi placentae.	Intervention study, three B-vitamins versus placebo (3000 pregnancies, periconceptional onset to end of pregnancy): plasma folic acid ↑, plasma HCys ↓, 63% fewer preeclampsia Risk classification for early abortions: HCys ≥9.9 µM → 2-fold; ≥12.3 µM → 4-fold; ≥15.3 µM → 7-fold [191]. Intervention study: 25 nullipara, MTHFR C677T-TT carriers, HCys > 12 μM, 3-5 early abortions; 5 mg folic acid and 750 mg vitamin B6 per day for 3 months → 22 women became pregnant without complications [192].
**Neural tube defects and other malformations—Rev** [193]	
High HCys levels: → Hypo-methylation of genomic DNA in the brain → Post-translational hypo-methylation of cytoskeletal proteins (see Figure 3) → Homocysteinylation of histone proteins (see Figure 4D) → disruption of gene expression. Recommended prevention with 0.4 mg folic acid/day → it takes 3 months to reach ≥900 mM erythrocyte folate [194].	Neural tube defects correlate directly with HCys and indirectly with folic acid and vitamin B12 levels in plasma as well as erythrocyte folate; increased risk in case erythrocyte folate <900 mM. MTHFR C677T-TT carriers → increased risk. Interventional studies with periconceptional folic acid substitution (0.4 mg/day)**—**meta-analysis: 72% reduction [195] → Interventional study with 0.8 mg folic acid, 4 μg B12, 2.6 mg B6/day versus placebo: >90% reduction and approx. 80% reduction in other defects (heart malformations, pyloric stenoses, ureteral obstruction) [196].
**Vitamin B deficiency in the mother****→ permanent disorders in the children—Rev** [197,198,199,200,201] Vitamin B12 ↓ → birth weight ↓, insulin resistance → visceral obesity, type 2 diabetes mellitus → metabolic syndrome, atherosclerosis and consequences	
Cause of the sequence listed above: B12 ↓ → HCys ↑ → methylation disorders with epigenetic effects in children (see Figure 3) B12-deficient diet (periconceptional until the end of pregnancy) in rats → offspring after one year: liver methylome with 190 differently methylated genes; liver proteome with 38 differently expressed proteins of lipid, carbohydrate and amino acid metabolism → atherogenic plasma lipid pattern (triglycerides ↑, HDL ↓) [202]. B12- and folic acid-free diet (periconceptional and during pregnancy and lactation) in rats → offspring after 80 days: pyramidal cell layer thickness (hippocampus) ↓, memory deficits [203]. High energy versus standard food with the same vitamin intake (3 years) in monkeys: high energy food results in more body fat and a lower birth weight of the offspring, plasma B12 ↓, atherogenic plasma lipid pattern	Meta-analysis of 20,000 women: plasma HCys in >90 percentile → 50% increased risk for children with reduced birth weight. Increased insulin resistance in 6-year-olds in case mothers had significantly low B12 levels during pregnancy.

**Table A9:** Infertility—**Rev** [201,204].

Plasma concentrations of vitamin B6, B12, folate and HCys are similar to those in seminal fluid. Every alteration of C1 metabolism associated with HCys ↑ leads to DNA fragmentation, telomere shortening, a different methylation pattern (see Figure 3) and radical formation (see Figure 4E) in sperm and oocytes. Hypomethylation of IGF2_H19 locus in men correlates with infertility. In vitro fertilization—quality of the embryo: Positive correlation with B12 content and negative correlation with HCys concentration in plasma and follicular fluid	Prospective study—approx. 20,000 women, 8 years: Infertility correlates negatively with daily folic acid intake. MTHFR C677T-TT carriers: more often infertile. Significantly higher levels of HCys in spermatozoa in infertile men. In vitro fertilization—intervention: Supplementation with the three B-vitamins reduces DNA fragmentation in sperm, doubles the pregnancy rate and triples the birth rate.

**Table A10:** Vision loss: exudative macular degeneration, diabetic retinopathy—**Rev** [205,206].

Human retinal cell culture: HCys induces production of VEGF (vascular endothelial growth factor). Plasma HCys correlates with VEGF-concentration in vitreous humor	Direct correlation between plasma HCys level and risk of macular degeneration. Significantly higher plasma HCys levels in exudative macular degeneration than in dry macular degeneration and controls. Intervention study: three B-vitamins versus placebo for 7 years in 5000 subjects: 34% less macular degeneration. Diabetes mellitus: in patients significantly higher HCys levels in serum, vitreous humor and retina.

**Table A11:** Increased fracture rate in old age—**Rev** [75].

S-homocysteinylation (see Figure 4D) of collagen fibrils hinders the regular formation of the bone matrix → increased fragility with mostly unchanged bone density.	Prospective studies: on average about twice the risk of femoral neck fractures with plasma HCys ≥15 μM. MTHFR C677T-TT carrier: significantly increased fracture rate. Interventional studies—three B-vitamins versus placebo: negative if related to bone density and plasma bone turnover parameters; mostly positive when it comes to fracture rates.

**Table A12:** Chronic fatigue syndrome, fibromyalgia—**Rev ** [207].

Chronic stress: (1) Increased formation of ROS (see Figure 4E) → peroxynitrite anion ↑ in the respiratory chain → irreversible inhibition of cytochrome C oxidase → cellular energy production ↓. (2) Leukocytes from patients with fibromyalgia: hypo-methylation and increased mRNA formation (see Figure 3) of genes with sensory, adrenergic and immunological functions. Cobalamin acts as an intracellular antioxidant in high concentrations.	Plasma vitamin B12 and HCys levels correlate positively/negatively, with exhaustion, comprehensive psychopathological rating scale, pain and memory ability. Therapy with high doses of vitamin B12 (1–2 mg/day) and folic acid (1–5 mg/day).

## Appendix B

The second part of the present (narrative) review might be categorized as systematized, but not as a systematic review, since it does not fulfill all aspects of the PRISMA guidelines, such as a comprehensive risk of bias assessment. Nevertheless, several characteristics of systematic reviews, e.g., a structured literature search, pre-defined exclusion criteria and the revision by a second investigator, have been considered.

### Appendix B.1. Literature Search Strategy

We systematically searched PubMed/Medline using a structured literature search filter. For that purpose, the animal-specific search filter template by Hooijmans and colleagues [208] has been adapted to the topic of this review with the aim to detect, as far as possible, all animal studies regarding HHCys in the context of cognitive decline, especially dementia-like disorders. In addition to the systematic record of studies, we manually included several hand-searched references from other sources for different reasons, such as the assessment of HCys in more exceptional biological matrices.

Overall, 154 studies have been included: the structured search resulted in 523 hits up to the year 2020, whereof titles and abstracts were screened. In the case of 19 hits, especially of the years 2018–2020, full texts were not available to us. 152 studies were selected for full text analysis. In this step, another 27 studies were excluded. Criteria for the inclusion of preclinical studies in the current review were the application of a method aiming to elevate HCys levels in animals. Furthermore, HCys levels and/or cognitive behavioral outcome should be reported by the authors. In addition to the systematically searched results, 29 additional references were included manually.

### Appendix B.2. Literature Analysis

All reviewed animal studies are listed in Table A13 and Table A14, serving as a basis for further analyses that are subsequently described in-depth.

Although this is no meta-analysis, we considered it interesting to provide a semi-quantitative impression of HCys levels depending on the different HHCys induction methods in order to enable a classification of the resulting hyperhomocysteinemic state in the animals. Therefore, the exact numbers of observations (n) in the single studies were not totaled here, but each study was considered as n = 1. A strict quantification of HCys levels was, furthermore, hardly possible because of limited comparability between the included studies due to a high diversity in the HHCys induction protocols:

Examples for this diversity were the type, duration and grade of deficiency in dietary induction methods, as well as in some cases a fasting period prior to the sampling step and the sampling method itself. The umbrella term “B-vit. def.” (B-vitamin deficiency) that we used (Table A13 and Table A14), mainly comprised folate (sometimes referred to as B9), B12 and to a lesser extent B6. Although it might be regarded as an independent HHCys inducing diet, we attributed lowered choline levels to the “B-vit. def.” category, since, in most of the reviewed studies, the impact of choline deficiency was investigated in combination with a lack of B-vitamins. In general, trials largely varied in the dietary restriction of vitamins (and vitamin-like substances), resulting in either mild, moderate or severe HHCys according to a frequently used classification system [209]. In the case that injections were used to elevate HCys levels, studies varied with respect to the time interval between injection and sampling step, which plays an important role in the assessment of (acute) HCys levels. Plasma sampling immediately after a single injection yields acutely higher HCys (e.g., [210]) than sampling after a longer period of time (e.g., [211]), due to excretion and degradation processes in the meantime. For several articles, an assignment to the aforementioned options was not possible because of an insufficient reporting. In the case of the common genetic induction methods, both homozygous and heterozygous models were subjects of investigation. A specialty was the maternal HHCys induction method, as it must be distinguished between dams and pups. In the case that a trial reported HCys levels for both dams and pups, the dam-related data were assigned to the primary HHCys induction method (in most cases a dietary regimen) and the pup-related were analyzed as “maternal HHCys impact”. Sex and age of the animals were additional sources of variance in HCys data between the studies. Last but not least, the same applied for the applied analytical method [209].

Several of the reviewed animal studies reported varying HCys levels, related to varying experimental conditions. In the case that HCys levels in a study were reported for wild type (WT) and transgenic (Tg) animals (e.g., [212]), only data derived from WT animals were included in the analysis for reasons of comparability. Data derived from Tg animals were only included in the analysis, if the transgenic model was a primary method to induce HHCys (e.g., deficiency of CBS, MTHFR…), but not if it was a transgenic model for another purpose (e.g., a model for early-onset Alzheimer’s disease). In the case that varying HCys levels in a single study derived from varying HHCys induction strategies, all of the reported levels were analyzed. Where applicable, data for males and females, as well as for different background strains in WT animals were pooled for reasons of comparability. In the case that an article reported data of more than one sampling step during a dietary HHCys induction period, only the full-length data were included in the analysis (e.g.,[213]). Since some of the reviewed studies did not report exact values for HCys levels in the text, levels were estimated from the related graphs in this case and marked in the tables. Others required a recalculation to µM in order to enable comparability with the other studies. In the case that recalculation to µM was not applicable (e.g., reporting of percentages or unfeasible units), levels of these studies were excluded from analysis. Insufficient reporting of HCys levels such as “*<5 µM*” or “*below quantification limit*” was also excluded from further analyses and led to divergent amounts of underlying studies for HHCys induction strategies (Figure 6C,D).

With respect to the analysis of behavioral experiments, we assigned the versatile cognitive tests of the reviewed studies to six cognitive domains: e.g., Morris water maze to “*spatial learning and memory*”, e.g., Y-maze to “*working memory*”, e.g., open field test to “*exploration and psychomotor function*”, e.g., novel object recognition test to “*recognition memory*”, e.g., elevated plus maze to “*anxiety and fear memory*”. Few tests that did not fit to one of these categories were grouped as “*others*”, but without being depicted in a separate graph due the high heterogeneity of this category. In some cases, transition between the categories is fluent, as e.g., the T-maze test might be attributed to working memory but also to explorative behavior. It was also challenging to assign particular behavioral tests to either “pure” motor function or cognitive performance. For example, locomotion in the open field test shows the distance moved by the animal and, therefore, is a marker of physical activity on the one hand. However. on the other hand, regarded over a period of time, locomotion is a parameter for habituation behavior, which is a form of learning [214]. The umbrella term “psychomotor” that we use in Table A13 is meant to comprise only behavioral testing with a relation to cognition such as exploration, sensorimotor testing or coordination tasks. Tests for motor function without a relation to cognitive performance such as testing of “pure” muscle function, e.g., paw grip endurance test, were not included in the analysis of cognitive outcomes. The assignment of behavioral tests to cognitive domains is in parts a subjective decision and might have been made differently by others.

**Table A13:** Reviewed animal studies derived from the systematic literature search of PubMed/Medline, which served as a basis for further analysis and indicates potential treatment options for elevated HCys levels or related symptoms; abbreviations: WT: wild type, KI: knock-in, KO: knock-out, Tg: transgenic, B-vit. def.: deficiency in B-vitamins (and related substances), Met suppl.: supplementation of L-methionine, CBS: cystathionine β-synthase, MTHFR: methylenetetrahydrofolate reductase, CTH: cystathionine γ-lyase.

		Strategy to Induce HHCys																			
		Diet/Drinking Water				Injection		Genetic Manipulation			Maternal HHCys Impact	Others		Investigated Biological Matrix						Impact on Cognitive Performance	
Publication	Animal Species	B-vit. def.	Met suppl.	HCys suppl.	Others	HCys	Others	CBS	MTHFR	Others			Blood Levels (µM): ↑HCys vs. Control/Baseline Data (Where Applicable)	Plasma	Serum	Brain Tissue	CSF	Urine	Liver Tissue	Cognitive Domain & Reported Effects of HHCys yes (+) or no (-)	Investigation of Potential Treatment Option
[83]	rat												28.8 vs. 6.3							spatial learning & memory (+)	ozagrel
[215]	rat												2.3 vs. 0.9 ^1^							spatial learning & memory (+)	edaravone
[80]	mouse												348.2 vs. 7.7 ^2;3;4^							exploration (-); anxiety (-); spatial learning & memory (-) ; recognition memory (-); others (-)	B-vitamins, PUFA, Fortasyn^®^ Connect-like diet
[216]	rat												5.65 vs. 4.85 (offspring)							offspring: working memory (+)	mild transient neonatal hypoxia
[217]	mouse												19.0 vs. < 5 (WT); 14.7 vs. < 5 (KI)							n.a.	n.a.
[218]	rat												11.22 vs. 7.08							n.a.	n.a.
[104]	rat												27.3 vs. 7.9 (dams); 19.5 vs. 6.3 (offspring)							offspring: exploration (+); anxiety (+); psychomotor function (+); working memory (+); spatial learning & memory (+); others (+)	sodium hydrosulfide
[219]	rat												n.a.							exploration (-); spatial learning & memory (+); fear memory (+)	synthetic tricyclic sulfonamide PP2A activators
[220]	mouse												n.a.							spatial learning & memory (+)	maternal choline supplementation
[123]	rat												10.1 vs. 6.1							exploration (-); recognition memory (+); spatial learning & memory (+)	emodin
[221]	rat												11.38 vs. 7.15							n.a.	n.a.
[222]	mouse												71.5 vs. 4.9							spatial learning & memory (+)	n.a.
[223]	mouse												423 vs. < 16							anxiety (n.a.); exploration (n.a.); others (+)	methionine restriction, enzyme replacement
[224]	mouse												140.50 vs. < 5							spatial learning & memory (+); others (-)	n.a.
[225]	mouse												22 vs. 17 (injection); 24 vs. 17 (age) ^1^							recognition memory (-); fear memory (+); spatial learning & memory (+)	B-vitamins, SAM
[101]	mouse												263 vs. 13 (CBS); 184 vs. 13 (CTH)							n.a.	n.a.
[226]	rat												13.13 vs. 8.5							spatial learning & memory (-); recognition memory (-); anxiety (-)	betaine
[227]	mouse												82.93 vs. 5.89 (WT); 84.67 vs. 6.34 (KO)							n.a.	n.a.
[105]	mouse												n.a.							exploration (+); anxiety (+); recognition memory (+); spatial learning & memory (+)	methionine restriction
[122]	rat												20 vs. 9 ^1^							spatial learning & memory (+)	liraglutide
[228]	mouse												13.97 vs. 8.55 (genetic); 18.93 vs. 8.55 (diet, WT); 38.87 vs. 13.97 (diet, Tg)							recognition memory (+); working memory (-); exploration (-); anxiety (+)	n.a.
[229]	rat												24 vs. 8 ^1^ (offspring)							offspring: sensorimotor function (+); spatial learning & memory (+)	n.a.
[230]	rat												17.5 vs. 8							n.a.	B-vitamins
[231]	rat												22 vs. 8 (Met suppl.); 62 vs. 8 (B-vit. def. + Met suppl.)							exploration (+); anxiety (+)	statins
[232]	mouse												n.a.							working memory (-); spatial learning & memory (+)	n.a.
[233]	rat												n.a.							spatial learning & memory (+)	Moringa oleifera extract
[113]	rat												28 vs. 10 ^1^							n.a.	epigallocatechin-3-gallate
[90]	rat												255.15 vs. 7.15 (acute); 16.64 vs. 7.15 (chronic)							n.a.	n.a.
[234]	mouse												52 vs. 22 ^1^							recognition memory (+)	n.a.
[235]	rat												0.59 vs. 0.3 ^1^							spatial learning & memory (+)	caffeine
[236]	mouse												22.01 vs. 14.43							anxiety (+); spatial learning & memory (+)	n.a.
[237]	rat												22 vs. 10 ^1^ (dams)							offspring: sensorimotor function (+); spatial learning & memory (+)	folate
[238]	rat												n.a.							exploration (-); spatial learning & memory (+); fear memory (+)	Ginkgo biloba extract
[239]	rat												n.a.							working memory (+); anxiety (+)	hydrogen sulfide
[118]	rat												n.a. ^2^							exploration (-); anxiety (-); spatial learning & memory (+); recognition memory (+)	n.a.
[111]	rat												9 vs. 4.5 ^1^							working memory (+)	Vitis vinifera leaves polyphenols
[102]	mouse												29 vs. 10 (homozygous); 11 vs. 10 (heterozygous) ^1^							spatial learning & memory (+); working memory (+); psychomotor function (-)	n.a.
[240]	rat												n.a.							recognition memory (+); fear memory (+)	creatine
[241]	rat												36 vs. 4 ^1^							spatial learning & memory (+); anxiety (+); exploration (-); psychomotor function (-)	hydrogen sulfide
[115]	rat												22 vs. 7 (diet); 12 vs. 7 (injection); 24 vs. 7 (diet + injection) ^1^							spatial learning & memory (+); recognition memory (+)	bosentan
[242]	mouse												n.a.							working memory (-); fear memory (-)	genetic absence of ALOX5
[243]	mouse												n.a.							working memory (+); fear memory (+); spatial learning & memory (+)	ALOX5 inhibition (zileuton)
[244]	rat												153.79 vs. 62.21 ^3^							working memory (+); spatial learning & memory (+)	fisetin
[245]	rat												165.48 vs. 49.64 ^3^							working memory (+); spatial learning & memory (+)	hesperidin
[246]	rat												n.a.							spatial learning & memory (+); recognition memory (+)	hydrogen sulfide
[247]	mouse												67.40 vs. < detection range (WT); 70.29 vs. < detection range (Tg)							spatial learning & memory (+)	anti-Aβ immunotherapy
[106]	rat												8.18 vs. 4.43 (diet); 7.37 vs. 4.43 (stress)							exploration (+); recognition memory (+); fear memory (+); spatial learning & memory (+)	B-vitamins, betaine
[248]	rat												26 vs. 15 (dams); 53 vs. 7 (offspring) ^1^							n.a.	maternal vitamin B6 supplementation
[249]	mouse												n.a.							recognition memory (+); fear memory (+)	hydrogen sulfide
[250]	mouse												n.a.							fear memory (+); spatial learning & memory (+)	n.a.
[251]	mouse												n.a.							spatial learning & memory (+); working memory (+); recognition memory (+)	cinnamon
[252]	mouse												46.1 vs. 4.6							spatial learning & memory (+)	Brazilian propolis extract
[253]	mouse												n.a. ^5^							working memory (+); fear memory (+)	betaine
[254]	mouse												22 vs. 14 (dams) ^1^ ; 28.4 vs. 9.8 (offspring)							offspring: recognition memory (+); working memory (-)	n.a.
[110]	rat												n.a. ^5^							exploration (+); others (+)	n.a.
[255]	rat												n.a.							spatial learning & memory (+)	atractylenolide III
[256]	mouse												18 vs. 13 (WT); 26 vs. 14 (Tg) ^1^							spatial learning & memory (+); psychomotor function (-); anxiety (+)	n.a.
[257]	rat												16.7 vs. 16.3							n.a.	zinc
[258]	rat												n.a.							fear memory (+); spatial learning & memory (+)	n.a.
[114]	rat												16 vs. 7 ^1^							spatial learning & memory (+); fear memory (+)	fatty acids
[259]	rat												n.a.							spatial learning & memory (+)	combination: acetylcholinesterase inhibitor + calcium channel blocker
[260]	rat												n.a.							offspring: exploration (+); anxiety (+); fear memory (+)	n.a.
[261]	mouse												22 vs. 6 ^1^							locomotion (-); recognition memory (-)	n.a.
[262]	mouse												n.a.							recognition memory (+)	ablation of MMP9 gene
[103]	mouse												13 vs. 3 (homozygous); 5 vs. 3 (heterozygous) ^1^							recognition memory (+); working memory (+)	n.a.
[263]	rat												n.a.							spatial learning & memory (+); recognition memory (+)	n.a.
[81]	rat												48 vs. 7 ^1^							exploration (-); anxiety (+); others (-)	n.a.
[264]	mouse												n.a.							working memory (-); fear memory (+); spatial learning & memory (+)	n.a.
[265]	rat												10 vs. 6 ^1^							spatial learning & memory (+)	hydroxysafflor yellow A
[94]	rat												n.a. ^2^							recognition memory (+)	memantine
[112]	rat												9.2 vs. 3.8							spatial learning & memory (+)	rivastigmine (liposomal)
[97]	mouse												7.5 vs. 5.5 (age); 11 vs. 5.5 (genetic, adult); 13.5 vs. 7.5 (genetic, old) ^1^							spatial learning & memory (+)	n.a.
[266]	mouse												26 vs. 8 (WT); 54 vs. 9 (Tg) ^1^							n.a.	n.a.
[267]	mouse												82.93 vs. 5.89							spatial learning & memory (+); psychomotor function (-)	n.a.
[268]	rat												n.a.							spatial learning & memory (+)	betaine
[269]	rat												19.16 vs. 5.21							spatial learning & memory (+)	resveratrol
[270]	rat												n.a.							spatial learning & memory (+)	n.a.
[98]	mouse												n.a.							psychomotor function (+); exploration (+); anxiety (+); recognition memory (+); working memory (+)	n.a.
[271]	rat												21.2 vs. 6.16							spatial learning & memory (+)	diethyl dithio carbamate trihydrate, folacin
[272]	rat												21 vs. 7.4 (dams)							offspring: spatial learning & memory (+)	ginkgo biloba extract
[273]	rat												5.1 vs. 3.2 ^1^							spatial learning & memory (+)	n.a.
[274]	rat												52.3 vs. 6.96							exploration (+); anxiety (+); others (+)	n.a.
[275]	mouse												90.68 vs. 2.04 (WT); 118.75 vs. 0.41 (Tg)							fear memory (-); spatial learning & memory (-)	SAM
[276]	rat												21.2 vs. 6.16							spatial learning & memory (+)	pioglitazone; rosiglitazone
[277]	mouse												100 vs. 8 (Met suppl.); 70 vs. 8 (B-vit. def.) ^1^							working memory (-); fear memory (-)	n.a.
[278]	rat												n.a.							spatial learning & memory (+); fear memory (+)	acetyl-L-carnitine
[279]	rat												n.a. ^2^							recognition memory (+); spatial learning & memory (+)	dextromethorphan
[78]	mouse												111 vs. 5 (WT); 76.4 vs. 3.8 (Tg)							n.a.	SAM
[280]	pig												6.88 vs. 5.45							exploration (+); psychomotor function (-); working memory (-); others (+)	folate
[109]	rat												n.a.							n.a.	N-acetyl cysteine + α-lipoic acid + α-tocopherol
[281]	rat												n.a.							fear memory (+); exploration (-)	curcumin
[282]	mouse												n.a.							spatial learning & memory (+)	n.a.
[210]	rat												∼500 µM vs. n.a.							n.a.	n.a.
[283]	mouse												2.39 vs. 2.37 (offspring)							offspring: exploration (-); anxiety (-)	n.a.
[87]	rat												26.7 vs. 10.4							spatial learning & memory (+)	n.a.
[130]	mouse												n.a. ^2^							spatial learning & memory (+)	anti-HCA antibody
[213]	mouse												16.8 vs. 3.4							fear memory (+)	n.a.
[284]	mouse												155 vs. 5 ^1^							n.a.	n.a.
[285]	mouse												30 vs. 6 ^1^							n.a.	n.a.
[286]	mouse												35.4 vs. 6.33							others (+)	n.a.
[287]	rat												n.a.							psychomotor function (-); fear memory (+)	folate
[288]	rat												16.5 vs. 6.8 (offspring)							offspring: sensorimotor function (+); spatial learning & memory (+); others (-)	short-term neonatal hypoxia
[289]	rat												10.2 vs. 6.2							spatial learning & memory (+)	B-vitamins
[92]	mouse												32.1 vs. 11.6							n.a.	n.a.
[290]	mouse												67 vs. 8.5 (WT); 49.9 vs. 9.6 (Tg)							exploration (+); spatial learning & memory (+); anxiety (+); others (+)	n.a.
[96]	mouse												257–365 vs. 15.4–25.4 (diff. strains)							exploration (-); anxiety (-); fear memory (+)	n.a.
[291]	rat												24.8 vs. 6.8 (dams)							offspring: spatial learning & memory (+)	melatonin
[292]	rat												31.3 vs. 4.2 (B-vit. def.); 31.2 vs. 4.2 (B-vit. def. + Met suppl.)							spatial learning & memory (+); psychomotor function (-)	methionine
[293]	mouse												28.7 vs. 5.2 (B-vit. def.); 13.9 vs. 5.2 (Met suppl.)							spatial learning & memory (+); psychomotor function (-)	n.a.
[79]	mouse												320 vs. 0.2 (WT); 450 vs. 1 (Tg) ^1^							spatial learning & memory (-)	n.a.
[294]	mouse												7.3 vs. 4.0							exploration (+); anxiety (+); working memory (-); psychomotor function (+); spatial learning & memory (-)	n.a.
[295]	rat												10.2 vs. 6.2 ^1^							n.a.	B-vitamins
[296]	rat												26 vs. 6 (dams)							offspring: spatial learning & memory (+)	n.a.
[203]	rat												13.3 vs. 6.8 (offspring)							offspring: sensorimotor function (-); anxiety (+); spatial learning & memory (+)	n.a.
[212]	mouse												101 vs. 37 (WT); 178 vs. 103 (Tg)							working memory (-); spatial learning & memory (+)	n.a.
[84]	mouse												243.7 vs. 5.1 (B-vit. def.); 86.7 vs. 5.1 (B-vit. def. + Met suppl.)							spatial learning & memory (+); psychomotor function (-); exploration (-)	B-vitamins
[297]	mouse												12.6 vs. 7.9							n.a.	n.a.
[211]	rat												4.5 vs. 2.9 ^1^							spatial learning & memory (-)	n.a.
[298]	rat												20 vs. 7.5 ^1^							fear memory (+); spatial learning & memory (+)	melatonin
[299]	mouse												205 vs. 3.9							n.a.	n.a.
[300]	rat												26.2 vs. 6.5							n.a.	folate
[301]	rat												400–500 vs. 10							spatial learning & memory (+); working memory (+); exploration (-)	n.a.
[302]	mouse												25 vs. 2 (WT); 27 vs. 3 (Tg) ^1^							n.a.	n.a.
[303]	mouse												5.3 vs. 3.25 (heterozygous); 32.3 vs. 3.25 (homozygous)							n.a.	n.a.
[304]	mouse												125 vs. 9							others (+)	n.a.

^1^: Estimated from graph; levels not exactly reported in the study; ^2^: HCA is also considered in the study; ^3^: data converted to µM; ^4^: for reasons of comparability with other studies: reporting of mean; not median as in the original manuscript; ^5^: transformation of data to µM not applicable.

**Table A14:** Additional hand-searched animal studies; abbreviations: WT: wild type, Tg: transgenic, B-vit. def.: deficiency in B-vitamins (and related substances), Met suppl.: supplementation of L-methionine, CBS: cystathionine β-synthase, MTHFR: methylenetetrahydrofolate reductase, GAA: guanidinoacetate.

		Strategy to Induce HHCys																	
		Diet/Drinking Water				Injection		Genetic Manipulation			Maternal HHCys Impact	Others		Investigated Biological Matrix					
Publication	Animal Species	B-vit. def.	Met suppl.	HCys suppl.	Others	HCys	Others	CBS	MTHFR	Others			Blood Levels (µM): ↑HCys vs. Control/Baseline Data (Where Applicable)	Plasma	Serum	Brain Tissue	CSF	Urine	Liver Tissue
[305]	mouse												6.5 vs. 5.1 ^1^ (offspring)						
[85]	mouse												243.7 vs. 4.6 (B-vit. def.); 86 vs. 4.6 (B-vit. def. + Met suppl.)						
[306]	mouse												349 vs. n.a.						
[307]	pig												72.33 vs. 10.53						
[308]	rat												34.1 vs. 15.1						
[309]	mouse												383.6 vs. n.a.						
[310]	mouse												19 vs. 10 (genetic); 16 vs. 10 (diet, WT);40 vs. 19 (diet, Tg) ^1^						
[311]	rat												45 vs. 15 (Met suppl.); 65 vs. 15 (GAA) ^1^						
[312]	mouse												9 vs. 1.5 ^1^						
[313]	mouse												51.8 vs. 3.0 (Met suppl.); 21.4 vs. 3.0 (HCys suppl.)						
[95]	rat												140 vs. 20 (diet); 68 vs. 15 (injection) ^1^						
[314]	mouse												3.8 vs. 3.7 (genetic); 40.7 vs. 3.7 (diet, WT); 140.3 vs. 6.8 (diet, Tg)						
[315]	mouse												23.5 vs. 4.1						
[209]	mouse												4.0 vs. 3.38						
[86]	mouse												4.5 vs. 3 (genetic); 4.4 vs. 3 (Met suppl., WT); 8.4 vs. 3 (B-vit. def., WT); 9.5 vs. 3 (Met suppl. + B-vit. def., WT) ^1^						
[316]	rabbit												20.3 vs. 12.3						
[317]	mouse												242 vs. 13						
[318]	mouse												8.2 vs. 4.0						
[319]	mouse												53.6 vs. 9.46 (Met suppl.); 51.4 vs. 9.46 (HCys suppl.)						
[107]	mouse												24.5 vs. 2.6						
[320]	rat												19.5 vs. 6.15						
[321]	rat												500 vs. n.a.						
[322]	mouse												8.3 vs. 5.0 (genetic); 17.2 vs. 5.0 diet, WT); 21.2 vs. 17.2 (diet, Tg)						
[323]	mouse												6.3 vs. 4.1 (genetic); 13.0 vs. 4.1 (diet, WT); 23.9 vs. 6.3 (diet, Tg)						
[88]	rat												15.5 vs. 10.5 ^1^						
[89]	rat												23.6 vs. 11.0						
[324]	monkey												10.6 vs. 4.0						
[325]	mouse												13.5 vs. 6.1 (heterozygous); 203.6 vs. 6.1 (homozygous)						
[326]	monkey												157 vs. 1						

^1^: Estimated from graph; levels not exactly reported in the study.
